# Dynamics of Ebola Disease in the Framework of Different Fractional Derivatives

**DOI:** 10.3390/e21030303

**Published:** 2019-03-21

**Authors:** Khan Muhammad Altaf, Abdon Atangana

**Affiliations:** 1 Department of Mathematics, City University of Science and Information Technology, Peshawar 25000, Pakistan; 2 Institute for Groundwater Studies, Faculty of Natural and Agricultural Sciences, University of the Free State, Bloemfontein 9300, South Africa

**Keywords:** Ebola model, Caputo derivative, Caputo–Fabrizio derivative, Atangana–Baleanu derivative, numerical results

## Abstract

In recent years the world has witnessed the arrival of deadly infectious diseases that have taken many lives across the globe. To fight back these diseases or control their spread, mankind relies on modeling and medicine to control, cure, and predict the behavior of such problems. In the case of Ebola, we observe spread that follows a fading memory process and also shows crossover behavior. Therefore, to capture this kind of spread one needs to use differential operators that posses crossover properties and fading memory. We analyze the Ebola disease model by considering three differential operators, that is the Caputo, Caputo–Fabrizio, and the Atangana–Baleanu operators. We present brief detail and some mathematical analysis for each operator applied to the Ebola model. We present a numerical approach for the solution of each operator. Further, numerical results for each operator with various values of the fractional order parameter α are presented. A comparison of the suggested operators on the Ebola disease model in the form of graphics is presented. We show that by decreasing the value of the fractional order parameter α, the number of individuals infected by Ebola decreases efficiently and conclude that for disease elimination, the Atangana–Baleanu operator is more useful than the other two.

## 1. Introduction

Ebola caused many deaths in Western Africa, especially in the outbreak of 2014. It includes more than 16 thousand laboratory cases with 70% death cases, which is regarded the deadliest outbreak in history since 1976 with 20 Ebola threats. It is evident that in each outbreak, the first case of infection occurred due to contact with infected animals such as monkeys, fruit bats, etc., which shows the spread of the virus through indirect contact [1]. It is documented in [2] that some percentage of the Ebola-Zaire type survived after two weeks on glass at 4 °C and (10%) on plastic, and on surfaces (3%). Moreover, 0.1% to 1 % of the Ebola virus particle can remain up to 50 days at 4 °C [3]. The survival of the Ebola virus in the environment due to poor sanitary and hygienic conditions considerably become another source of Ebola infection in Africa. In Africa, regions were affected greatly by the Ebola virus outbreak due to their inhabitants being involved in hunting food, being close to the rain-forest, and harvesting forest fruits for food [4,5].

The Ebola disease outbreaks and their transmission have been documented in many articles (see [6,7,8,9,10] and the references therein) and the main focus was to study the human population and the direct transmission. Some models of the type SI, SIR, SEIR, and other types also considered the dynamics of the Ebola disease outbreaks [9,11,12]. Recently, in [13] studied an Ebola virus disease through a simple mathematical model of the type SIR with the inclusion of environment effect. Due to the fact that Ebola virus survives in the environment, this warrants that future epidemics can occur. Thus, the inclusion of the environment effect in Ebola disease spread should be studied more and some preventive and other measures should be used to protect people further from this deadly infection. Therefore, based on the model presented in [13], we aim to study the Ebola disease model in the framework of the fractional calculus. The reason for the use of the fractional calculus in Ebola disease is that it has many advantages. Some of them are the heredity and memory effects, the parameter estimations are better, the crossover behavior of the model, and effective strategies for the case of arbitrary order. Some other works used it to study the dynamics of complex networks [14,15,16]. In [14] the authors studied the dynamics of information and the uses in complex networks. Coupling dynamics of an epidemic spreading with information diffusion is analyzed in [15]. The events that determine spreading dynamics and the information transmission through internal and external influences are considered in [16].

Fractional calculus and its applications to real life problems is found extensively in the literature, for example [17,18,19,20,21]. In all these mentioned papers the focus is to eliminate the infection from the community and it is proven that the fractional models have the ability to model such epidemic disease efficiently and provide reasonable results for the case of non-integer. It is shown that the fractional models are useful for the data fitting [22]. The results suggest in [22] that fractional models are efficient to study disease dynamics well. Therefore, motivated with the above applications, we aim to study an Ebola disease model in the fractional order. We consider three different fractional operators, that is, the Caputo, Caputo–Fabrizio, and the Atangana–Baleanu derivatives. According to the authors’ knowledge no one has applied the three operators to an epidemic model. So, this work is a useful study to analyze Ebola disease with different fractional operators. The rest of the work on Ebola disease is categorized as follows: The fractional background material are shown in Section 2. A mathematical model on Ebola disease is presented in Section 3 with basic mathematical results. In Section 4, a mathematical model in the frame of the Caputo derivative and their numerical results, the Caputo–Fabrizio derivative is used to formulate the model and their relevant results are presented, and we further consider the Atangana–Baleanu model for Ebola disease and discuss its existence and uniqueness and a useful numerical scheme for their solution, and lastly in this section, the comparison results for these operators with various fractional order parameters are shown. The Ebola disease models and their fractional results are summarized in Section 5.

## 2. Fundamental Concepts

Here, we recall the fundamental concepts regarding the Caputo, Caputo–Fabrizio, and the Atangana–Baleanu derivative.

**Definition** **1.** *For a function*$f:R^{+}\to R$*, then the fractional integral of order*α>0*is given by*

$$\begin{array}{ccc} I_{t}^{\alpha }\left(f\left(t\right)\right) & = & \frac{1}{\Gamma (\alpha )}\int_{0}^{t}{\left(t-z\right)}^{\alpha -1}f\left(z\right)dz. \end{array}$$

*where* Γ *shows the Gamma function and α is the fractional order parameter.*

**Definition** **2.** *For a function* $f\in C^{n}$ *, then the Caputo derivative with order α is defined as*

$$\begin{array}{c} {}^{C}D_{t}^{\alpha }\left(f\left(t\right)\right)=I^{n-\alpha }D^{n}f\left(t\right)=\frac{1}{\Gamma (n-\alpha )}\int_{0}^{t}\frac{f^{n}\left(z\right)}{{\left(t-z\right)}^{\alpha +n-1}}dz, \end{array}$$

*that is defined for the absolute continuous functions and* n−1<α<n∈N *. Obviously,* ${}^{C}D_{t}^{\alpha }\left(f\left(t\right)\right)$ *tends to* $f^{\prime }\left(t\right)$ *as* α→1 *.*

**Definition** **3.** *[23]. Let* $z\in H^{1}\left(a,b\right)$ *, with* b>a *, and* 0≤α≤1 *, then the Caputo–Fabrizio derivative can be written as*

(1) $$\begin{array}{c} D_{t}^{\alpha }\left(z\left(t\right)\right)=\frac{K(\alpha )}{1-\alpha }\int_{a}^{t}z^{\prime }\left(x\right)\exp\left[-\alpha \frac{t-x}{1-\alpha }\right]dx, \end{array}$$

*the normalized function is shown by* K(α) *and it holds* K(0)=K(1)=1 *. Consider the case for which* $z\notin H^{1}\left(a,b\right)$ *then, we have the following:*

(2) $$\begin{array}{c} D_{t}^{\alpha }\left(z\left(t\right)\right)=\frac{\alpha \;K(\alpha )}{1-\alpha }\int_{a}^{t}\left(z\left(t\right)-z\left(x\right)\right)\exp\left[-\alpha \frac{t-x}{1-\alpha }\right]dx. \end{array}$$

**Remark** **1.** *[24]. Let $\nu =\frac{1-\alpha }{\alpha }\in \left[0,\infty \right)$, $\alpha =\frac{1}{1+\nu }\in \left[0,1\right]$, then equation given by (2) can be expressed is as follows,*

(3) $$\begin{array}{c} D_{t}^{\nu }\left(z\left(t\right)\right)=\frac{K(\nu )}{\nu }\int_{a}^{t}z^{\prime }\left(x\right)\exp\left[-\frac{t-x}{\nu }\right]dx,\;K\left(0\right)=K\left(\infty \right)=1. \end{array}$$

*Further,*

(4) $$\begin{array}{c} \lim_{\nu ⟶0}\frac{1}{\nu }\exp\left[-\frac{t-x}{\nu }\right]=\varphi \left(x-t\right). \end{array}$$

**Definition** **4.** *Consider* α∈(0,1) *, for a function* z(x) *then we can write the integral of fractional order α is as follows,*

(5) $$\begin{array}{c} I_{t}^{\alpha }\left(z\left(t\right)\right)=\frac{2(1-\alpha )}{\left(2-\alpha )K(\alpha \right)}g\left(t\right)+\frac{2\alpha }{\left(2-\alpha )K(\alpha \right)}\int_{0}^{t}z\left(s\right)ds,t\geq 0. \end{array}$$

**Remark** **2.** *In Equation (4), the remainder of the Caputo type non-integer order integral of the function with order* α∈(0,1) *is a mean into z with integral of order 1. Thus, it requires,*

(6) $$\begin{array}{c} \frac{2}{2K(\alpha )-\alpha K(\alpha )}=1, \end{array}$$

*implies that* $K\left(\alpha \right)=\frac{2}{2-\alpha }$ *,* α∈(0,1) *. Based on Equation (6), a new Caputo derivative is suggested with* α∈(0,1) *and is given by*

(7) $$\begin{array}{c} D_{t}^{\alpha }\left(z\left(t\right)\right)=\frac{1}{1-\alpha }\int_{0}^{t}z^{\prime }\left(x\right)\exp\left[-\alpha \frac{t-x}{1-\alpha }\right]dx. \end{array}$$

In the following we present the new derivative known as the Atangana–Baleanu derivatives having non-singular and non-local kernel [25].

**Definition** **5.** *Let* $f\in H^{1}\left(a,b\right)$ *,* b>a *,* α∈[0,1], *then in the Caputo sense the Atangana–Baleanu derivative is defined as:*

(8) $$\begin{array}{ccc} {}_{a}^{ABC}D_{t}^{\alpha }f\left(t\right) & = & \frac{K(\alpha )}{1-\alpha }\int_{a}^{t}f^{\prime }\left(z\right)E_{\alpha }\left[-\alpha \frac{{\left(t-z\right)}^{\alpha }}{1-\alpha }\right]dz. \end{array}$$

**Definition** **6.** *The fractional integral associated with the Atangana–Beleanu derivative is given by:*

(9) $$\begin{array}{ccc} {}_{a}^{ABC}I_{t}^{\alpha }f\left(t\right) & = & \frac{1-\alpha }{K(\alpha )}f\left(t\right)+\frac{\alpha }{K(\alpha )\Gamma (\alpha )}\int_{a}^{t}f\left(z\right){\left(t-z\right)}^{\alpha -1}dz. \end{array}$$

*when the fractional order turns to zero, we can obtain the original function.*

**Theorem** **1.** *Consider the function* f∈C[a,b] *, then the following holds [25]:*

(10) $$\begin{array}{c} ∥{}_{a}^{ABC}D_{t}^{\alpha }\left(f\left(t\right)\right)∥<\frac{K(\alpha )}{1-\alpha }∥f\left(t\right)∥,\;where\;∥f\left(t\right)∥=max_{a\leq t\leq b}\left|f\left(t\right)\right|. \end{array}$$

*Further, for the newly derivative the Lipschitz condition can be easily satisfied [25]:*

(11) $$\begin{array}{c} ∥{}_{a}^{ABC}D_{t}^{\alpha }f_{1}\left(t\right)-{}_{a}^{ABC}D_{t}^{\alpha }f_{2}\left(t\right)∥<\varpi_{1}∥f_{1}\left(t\right)-f_{2}\left(t\right)∥. \end{array}$$

**Theorem** **2.** *A given fractional differential equation:*

(12) $${}_{a}^{ABC}D_{t}^{\alpha }f\left(t\right)=s\left(t\right),$$

*has the unique solution given by [25]:*

(13) $$\begin{array}{c} f\left(t\right)=\frac{1-\alpha }{K(\alpha )}s\left(t\right)+\frac{\alpha }{K(\alpha )\Gamma (\alpha )}\int_{a}^{t}s\left(z\right){\left(t-z\right)}^{\alpha -1}dz. \end{array}$$

## 3. Model Formulation

We begin to formulate the Ebola epidemic disease by considering the human population in three compartments, that is, the susceptible individuals, S(t), individuals infected with Ebola virus, I(t), and the individuals recovered from the Ebola virus, R(t). The individuals infected with Ebola and the deceased is D(t) and P(t) is the class for the Ebola virus pathogen in the environment. The model that describes the dynamics of Ebola disease modeled through differential equations is given by

(14) $$\left\{\begin{array}{ccc} \frac{dS}{dt} & = & \Lambda -\lambda S-dS,\\ \frac{dI}{dt} & = & \lambda S-(d+\delta +\phi_{1})I,\\ \frac{dR}{dt} & = & \phi_{1}I-dR,\\ \frac{dD}{dt} & = & (d+\delta )I-\varepsilon D,\\ \frac{dP}{dt} & = & \varpi +\xi I+\theta D-\kappa P, \end{array}\right.$$

where $\lambda =\beta_{1}I+\beta_{2}D+\psi P$, and the appropriate initial conditions are given by

(15) $$\begin{array}{c} S\left(0\right)=S_{0},\;I\left(0\right)=I_{0},\;R\left(0\right)=R_{0},\;D\left(0\right)=D_{0},\;P\left(0\right)=P_{0}. \end{array}$$

The birth rate of the susceptible individuals is recruited by the rate Λ, while the death rate is given by *d*. The susceptible individuals become infectious with the effective contact rate $\beta_{1}$ and $\beta_{2}$ with the deceased human individuals. The susceptible are able to attract the disease from the contaminated environment at a rate given by ψ. The death rate of the infected individuals due to Ebola virus is given by a rate δ, while the recovery from infection is $\phi_{1}$. The deceased people can be directly buried during funerals at rate ε. At a rate of ϖ the environment is contaminated by the Ebola virus. At rates of ξ and θ the infected and deceased individuals, respectively, shed the virus in the environment. The virus decay of the Ebola virus from the population is given by parameter κ.

The sum of the first three equations of the Ebola disease model Equation (14) is given by

(16) $$\begin{array}{ccc} \frac{dN}{dt} & = & \Lambda -dN-\delta I, \end{array}$$

where N=S+I+R denotes the total alive human population. It should be noted that ε≤(d+δ), which is an appropriate condition for the compartment *D* for which the model becomes relevant, otherwise the deceased human individuals will disappear and the model would be irrelevant. Further, the model given by Equation (14) is well posed and biologically feasible in the region given by

(17) $$\begin{array}{c} \Phi =\left\{M\in R_{+}^{5}:N\left(t\right)\leq \frac{\Lambda }{d},\;D\leq \frac{(d+\delta )\Lambda }{\varepsilon d},P\left(t\right)=\frac{\varepsilon (d\varpi +\Lambda \xi )+\theta \Lambda (\delta +d)}{d\kappa \varepsilon }\right\}, \end{array}$$

where M=(S(t),I(t),R(t),D(t),P(t)).

## 4. Ebola Model in the Caputo Sense

The purpose of this section is to apply the proposed three operators on the Ebola disease model Equation (14). Initially, we will apply the Caputo derivative on the Ebola disease model, then, the Caputo–Fabrizio derivative, and finally the Atangana–Baleanu derivative. For each operator we will provide the solution procedure and the discussion on the graphical results in details. So, we start with the Caputo sense.

### 4.1. Ebola Model in the Caputo Sense

We can express the model given by Equation (14) in the Caputo derivative as follows:

(18) $$\left\{\begin{array}{ccc} {}_{0}^{C}D_{t}^{\alpha }S & = & \Lambda -\lambda S-dS,\\ {}_{0}^{C}D_{t}^{\alpha }I & = & \lambda S-(d+\delta +\phi_{1})I,\\ {}_{0}^{C}D_{t}^{\alpha }R & = & \phi_{1}I-dR,\\ {}_{0}^{C}D_{t}^{\alpha }D & = & (d+\delta )I-\varepsilon D,\\ {}_{0}^{C}D_{t}^{\alpha }P & = & \varpi +\xi I+\theta D-\kappa P, \end{array}\right.$$

where $\lambda =\beta_{1}I+\beta_{2}D+\psi P$, and with the initial conditions, $S\left(0\right)=S_{0},\;I\left(0\right)=I_{0},\;R\left(0\right)=R_{0},\;D\left(0\right)=D_{0}$, and $P\left(0\right)=P_{0}$.

### 4.2. Equilibrium Points

For the Ebola disease model Equation (18) in the Caputo sense, there is no disease-free equilibrium when ϖ>0 and we have the other equilibrium say, $E^{*}=\left(S^{*},I^{*},R^{*},D^{*},P^{*}\right)$, we have

$$\begin{array}{c} \left\{\begin{array}{c} S^{*}=\frac{\Lambda -\left(d+\delta +\phi_{1}\right)I^{*}}{d},\\ R^{*}=\frac{\phi_{1}I^{*}}{d},\\ D^{*}=\frac{\left(d+\delta \right)I^{*}}{\epsilon },\\ P^{*}=\frac{\theta I^{*}\left(d+\delta \right)+I^{*}\xi \epsilon +\varpi \varepsilon }{\kappa \varepsilon } \end{array}\right. \end{array}$$

Using these values in the second equation of the model Equation (18), we have

(19) $$\begin{array}{c} C_{2}{I^{*}}^{2}-C_{1}I^{*}-C_{0}=0, \end{array}$$

where

$$\begin{array}{ccc} C_{2} & = & \left(d+\delta +\phi_{1}\right)\left(\beta_{1}\kappa \varepsilon +\beta_{2}\kappa \left(d+\delta \right)+\psi \left(\theta \left(d+\delta \right)+\xi \varepsilon \right)\right),\\ C_{1} & = & -(-\kappa \Lambda \left(\beta_{1}\varepsilon +\beta_{2}\left(d+\delta \right)\right)-\psi \left(\theta \Lambda \left(d+\delta \right)-\varpi \varepsilon \left(d+\delta \right)+\Lambda \xi \varepsilon \right)+d\kappa \varepsilon \left(d+\delta \right))\\ & & -\varepsilon \phi_{1}\left(d\kappa +\varpi \psi \right),\\ C_{0} & = & \Lambda \varpi \psi \varepsilon . \end{array}$$

We have from the coefficient $C_{1}$,

$$\begin{array}{c} C_{1}=d\varepsilon \kappa \left(d+\delta +\phi_{1}\right)\left(R_{0}-1-\frac{\psi \varpi }{d\kappa }\right), \end{array}$$

where

$$\begin{array}{c} R_{0}=\frac{\Lambda \beta_{1}}{d(d+\delta +\phi_{1})}+\frac{\Lambda \beta_{2}\left(d+\delta \right)}{d\varepsilon (d+\delta +\phi_{1})}+\frac{\psi \Lambda (\varepsilon \xi +\theta \delta +\theta d)}{d\varepsilon \kappa (d+\delta +\phi_{1})}. \end{array}$$

Considering the case when ϖ=0, we have

$$\begin{array}{c} \left\{\begin{array}{c} S^{*}=\frac{\Lambda }{dR_{0}},\\ I^{*}=\frac{\Lambda (R_{0}-1)}{\left(d+\delta +\phi_{1}\right)R_{0}},\\ R^{*}=\frac{\Lambda \phi_{1}\left(R_{0}-1\right)}{d\left(d+\delta +\phi_{1}\right)R_{0}},\\ D^{*}=\frac{\left(\Lambda \left(d+\delta \right)\right)\left(R_{0}-1\right)}{\varepsilon \left(d+\delta +\phi_{1}\right)R_{0}},\\ P^{*}=\frac{\Lambda \left(\varepsilon \xi +\theta \left(d+\delta \right)\right)\left(R_{0}-1\right)}{\varepsilon \kappa \left(d+\delta +\phi_{1}\right)R_{0}}, \end{array}\right. \end{array}$$

and we have a disease-free equilibrium,

$$\begin{array}{c} E_{0}=\left(\frac{\Lambda }{d},0,0,0\right), \end{array}$$

known as Ebola virus-free equilibrium.

### 4.3. Numerical Procedure for the Ebola Disease Model in the Caputo Sense

In the present subsection, we present the numerical scheme for the solution of the fractional Ebola disease model in the Caputo sense Equation (18). The present scheme that we use for the solution of the fractional Caputo nonlinear ordinary differential equation has been presented in [26,27]. The following procedure is presented

(20) $$\begin{array}{c} {}_{0}^{C}D_{t}^{\alpha }z\left(t\right)=f\left(t,z\left(t\right)\right). \end{array}$$

Using the fundamental theorem on Equation (20), we obtain

(21) $$\begin{array}{c} z\left(t\right)-z\left(0\right)=\frac{1}{\Gamma (\alpha )}\int_{0}^{t}f\left(\chi ,z\left(\chi \right)\right){\left(t-\chi \right)}^{\alpha -1}d\chi , \end{array}$$

thus, at $t=t_{n+1}$, n=0,1,…, the following is obtained

(22) $$\begin{array}{c} z\left(t_{n+1}\right)-z\left(0\right)=\frac{1}{\Gamma (\alpha )}\int_{0}^{t_{n+1}}{\left(t_{n+1}-t\right)}^{\alpha -1}f\left(t,z\left(t\right)\right)dt, \end{array}$$

and

(23) $$\begin{array}{c} z\left(t_{n}\right)-z\left(0\right)=\frac{1}{\Gamma (\alpha )}\int_{0}^{t_{n}}{\left(t_{n}-t\right)}^{\alpha -1}f\left(t,z\left(t\right)\right)dt. \end{array}$$

From Equations (23) and (22), we have

(24) $$\begin{array}{ccc} z(t_{n+1}) & = & z\left(t_{n}\right)+\underset{A_{\alpha ,1}}{\underset{︸}{\frac{1}{\Gamma (\alpha )}\int_{0}^{t_{n+1}}{\left(t_{n+1}-t\right)}^{\alpha -1}f\left(t,z\left(t\right)\right)dt}}\\ & & -\underset{A_{\alpha ,2}}{\underset{︸}{\frac{1}{\Gamma (\alpha )}\int_{0}^{t_{n}}{\left(t_{n}-t\right)}^{\alpha -1}f\left(t,z\left(t\right)\right)dt}}. \end{array}$$

where

(25) $$\begin{array}{c} A_{\alpha ,1}=\frac{1}{\Gamma (\alpha )}\int_{0}^{t_{n+1}}{\left(t_{n+1}-t\right)}^{\alpha -1}f\left(t,z\left(t\right)\right)dt, \end{array}$$

and

(26) $$\begin{array}{c} A_{\alpha ,2}=\frac{1}{\Gamma (\alpha )}\int_{0}^{t_{n}}{\left(t_{n}-t\right)}^{\alpha -1}f\left(t,z\left(t\right)\right)dt. \end{array}$$

Using the Lagrange approximation for the function f(t,z(t)), we have

(27) $$\begin{array}{ccc} P(t) & ≃ & \frac{t-t_{n-1}}{t_{n}-t_{n-1}}f\left(t_{n},z_{n}\right)+\frac{t-t_{n}}{t_{n-1}-t_{n}}f\left(t_{n-1},z_{n-1}\right)\\ & = & \frac{f(t_{n},z_{n})}{h}\left(t-t_{n-1}\right)-\frac{f(t_{n-1},z_{n-1})}{h}\left(t-t_{n}\right). \end{array}$$

The use of the above expression leads to

(28) $$\begin{array}{ccc} A_{\alpha ,1} & = & \frac{f(t_{n},z_{n})}{h\Gamma (\alpha )}\int_{0}^{t_{n+1}}{\left(t_{n+1}-t\right)}^{\alpha -1}\left(t-t_{n-1}\right)dt\\ & & -\frac{f(t_{n-1},z_{n-1})}{h\Gamma (\alpha )}\int_{0}^{t_{n+1}}{\left(t_{n+1}-t\right)}^{\alpha -1}\left(t-t_{n}\right)dt. \end{array}$$

We have, after further simplification

(29) $$\begin{array}{ccc} A_{\alpha ,1} & = & \frac{f(t_{n},z_{n})}{h\Gamma (\alpha )}\left[\frac{2h}{\alpha }t_{n+1}^{\alpha }-\frac{t_{n+1}^{\alpha +1}}{\alpha +1}\right]\\ & & -\frac{f(t_{n-1},z_{n-1})}{h\Gamma (\alpha )}\left[\frac{h}{\alpha }t_{n+1}^{\alpha }-\frac{1}{\alpha +1}t_{n+1}^{\alpha +1}\right]. \end{array}$$

Similarly,

(30) $$\begin{array}{ccc} A_{\alpha ,2} & = & \frac{1}{\Gamma (\alpha )}\int_{0}^{t_{n}}{\left(t_{n}-t\right)}^{\alpha -1}[\frac{f(t_{n},z_{n})}{h}\left(t-t_{n-1}\right)\\ & & -\frac{f(t_{n-1},z_{n-1})}{h}\left(t-t_{n}\right)]dt. \end{array}$$

Further simplifying, we get

(31) $$\begin{array}{ccc} A_{\alpha ,2} & = & \frac{f(t_{n},z_{n})}{h\Gamma (\alpha )}\left[\frac{h}{\alpha }t_{n}^{\alpha }-\frac{t_{n}^{\alpha +1}}{\alpha +1}\right]\\ & & +\frac{f(t_{n-1},z_{n-1})}{h\Gamma (\alpha )}\left[\frac{1}{\alpha +1}t_{n}^{\alpha +1}\right]. \end{array}$$

We have the final approximate solution for the fractional nonlinear ordinary differential equation by substituting the Equations (30) and (31) into (24), given by

(32) $$\begin{array}{ccc} z(t_{n+1}) & = & z\left(t_{n}\right)+\frac{f(t_{n},z_{n})}{h\Gamma (\alpha )}\left[\frac{2ht_{n+1}^{\alpha }}{\alpha }-\frac{t_{n+1}^{\alpha +1}}{\alpha +1}+\frac{h}{\alpha }t_{n}^{\alpha }-\frac{t_{n+1}^{\alpha +1}}{\alpha +1}\right]\\ & & +\frac{f(t_{n-1},z_{n-1})}{h\Gamma (\alpha )}\left[-\frac{h}{\alpha }t_{n+1}^{\alpha }+\frac{t_{n+1}^{\alpha +1}}{\alpha +1}+\frac{t_{n}^{\alpha +1}}{\alpha +1}\right]. \end{array}$$

**Figure 1:**
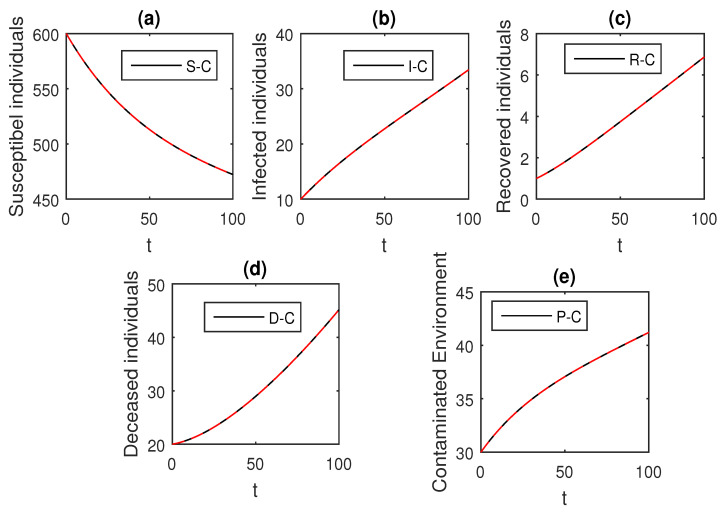
The graphical results show the dynamics of the Caputo derivative model (18), when α=1, where (**a**) Susceptible individuals, (**b**) Infected individuals, (**c**) recovered individuals, (**d**) deceased individuals, (**e**) Environment pathogens.

**Figure 2:**
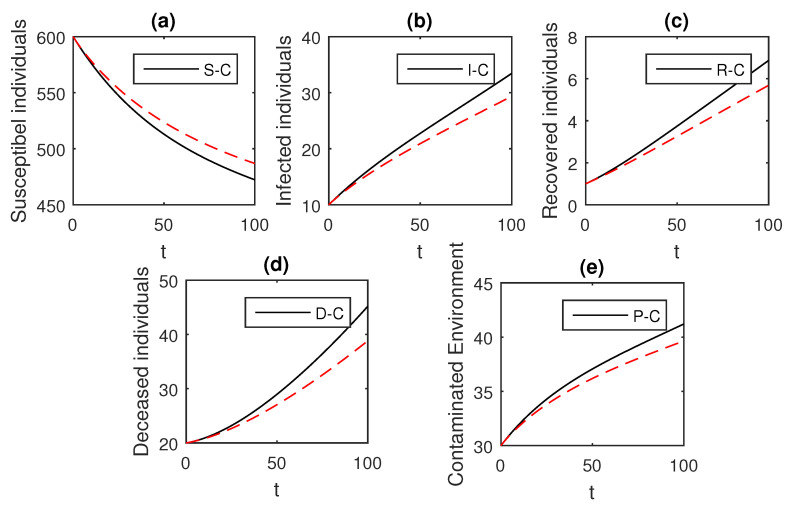
The graphical results show the dynamics of the Caputo derivative model (18), when α=0.95, where (**a**) Susceptible individuals, (**b**) Infected individuals, (**c**) recovered individuals, (**d**) deceased individuals, (**e**) Environment pathogens.

**Figure 3:**
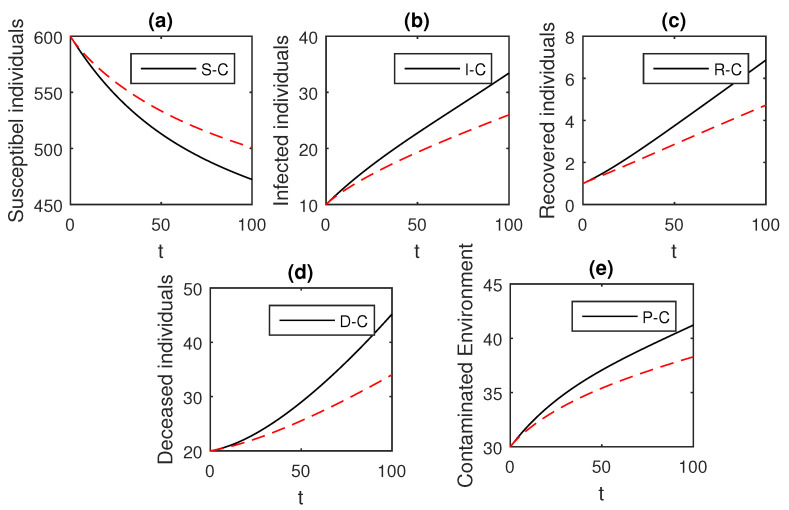
The graphical results show the dynamics of the Caputo derivative model (18), when α=0.9, where (**a**) Susceptible individuals, (**b**) Infected individuals, (**c**) recovered individuals, (**d**) deceased individuals, (**e**) Environment pathogens.

**Figure 4:**
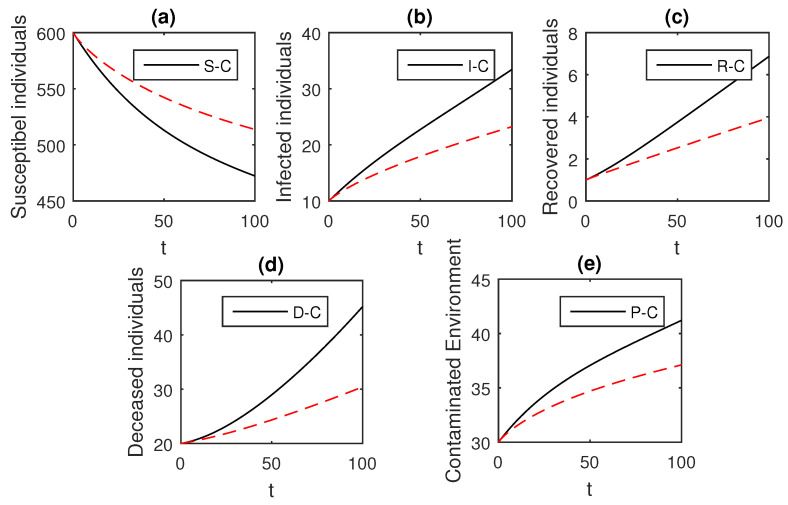
The graphical results show the dynamics of the Caputo derivative model (18), when α=0.85, where (**a**) Susceptible individuals, (**b**) Infected individuals, (**c**) recovered individuals, (**d**) deceased individuals, (**e**) Environment pathogens.

**Figure 5:**
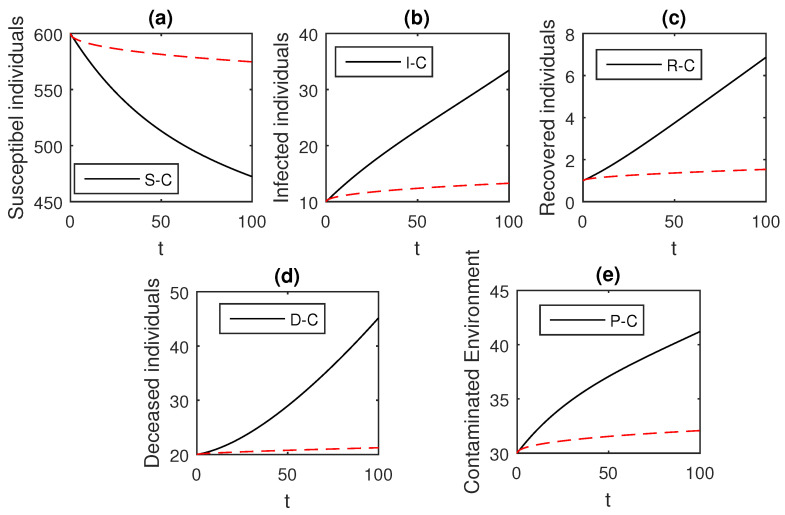
The graphical results show the dynamics of the Caputo derivative model (18), when α=0.5, where (**a**) Susceptible individuals, (**b**) Infected individuals, (**c**) recovered individuals, (**d**) deceased individuals, (**e**) Environment pathogens.

**Figure 6:**
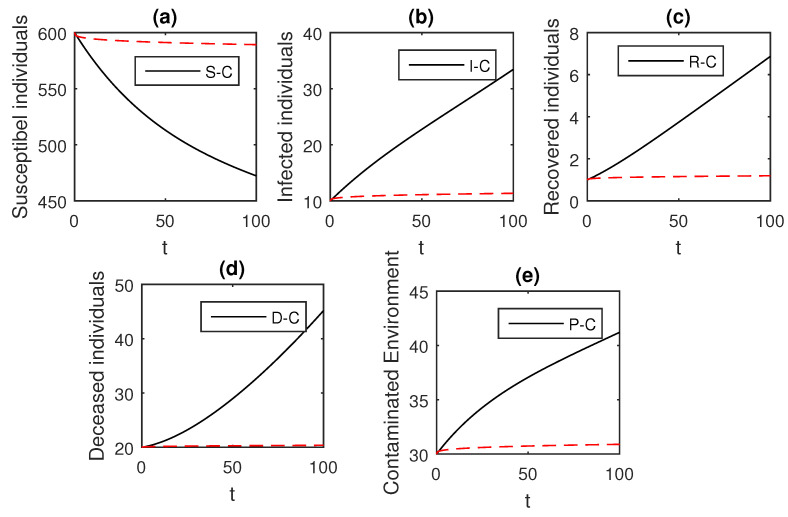
The graphical results show the dynamics of the Caputo derivative model (18), when α=0.3, where (**a**) Susceptible individuals, (**b**) Infected individuals, (**c**) recovered individuals, (**d**) deceased individuals, (**e**) Environment pathogens.

**Figure 7:**
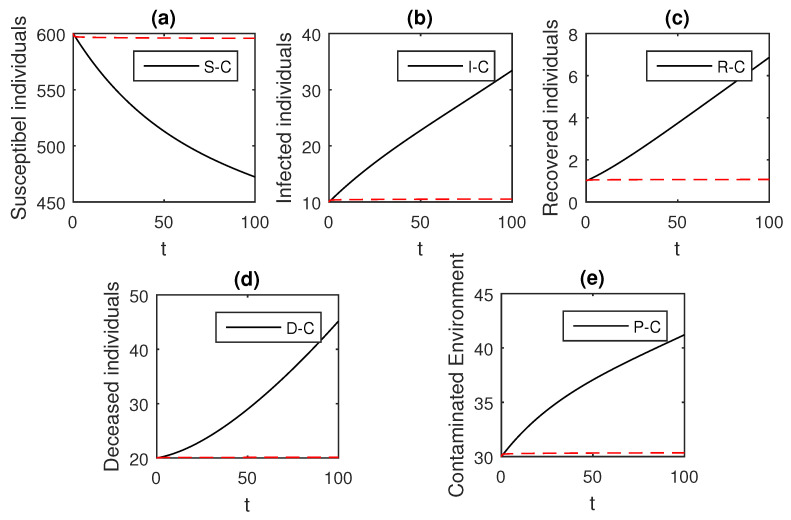
The graphical results show the dynamics of the Caputo derivative model (18), when α=0.1, where (**a**) Susceptible individuals, (**b**) Infected individuals, (**c**) recovered individuals, (**d**) deceased individuals, (**e**) Environment pathogens.

The above scheme is used further for the solution of the Ebola disease model in the Caputo sense Equation (18) by considering the parameter values, d=0.05, δ=0.05, ϕ=0.06, Λ=10, ϵ=0.008, ξ=0.004, κ=0.03, ψ=0.01, $\beta_{1}=0.006$, $\beta_{2}=0.012$, ϖ=1, and θ=0.004, and with various values of the fractional order parameter α. We have the graphical results for the numerical solution of the Ebola disease model Equation (18) in Figure 1, Figure 2, Figure 3, Figure 4, Figure 5, Figure 6 and Figure 7. One can observe in Figure 1, Figure 2, Figure 3, Figure 4, Figure 5, Figure 6 and Figure 7 by deceasing the value of α, the individuals infected with Ebola decreases while the population of infected individuals increases. We use this further to check the graphical results for the case when α=0.3,0.1, then, one can see that infection is almost on the steady state, see Figure 6 and Figure 7.

### 4.4. Ebola Model in the Caputo–Fabrizio Sense

We can express the model given by Equation (14) in Caputo–Fabrizio derivative as follows:

(33) $$\left\{\begin{array}{ccc} {}_{0}^{CF}D_{t}^{\alpha }S & = & \Lambda -\lambda S-dS,\\ {}_{0}^{CF}D_{t}^{\alpha }I & = & \lambda S-(d+\delta +\phi_{1})I,\\ {}_{0}^{CF}D_{t}^{\alpha }R & = & \phi_{1}I-dR,\\ {}_{0}^{CF}D_{t}^{\alpha }D & = & (d+\delta )I-\varepsilon D,\\ {}_{0}^{CF}D_{t}^{\alpha }P & = & \varpi +\xi I+\theta D-\kappa P, \end{array}\right.$$

where $\lambda =\beta_{1}I+\beta_{2}D+\psi P$, and with the initial conditions, $S\left(0\right)=S_{0},\;I\left(0\right)=I_{0},\;R\left(0\right)=R_{0},\;D\left(0\right)=D_{0}$, and $P\left(0\right)=P_{0}$.

### 4.5. Numerical Solution for Caputo–Fabrizio Model

Here we present the numerical solution for the Caputo–Fabrizio model Equation (33) by using the scheme presented [27]. The following steps are taken as specified in one for the solution of Equation (33).

(34) $$\begin{array}{ccc} S(t)-S(0) & = & \frac{\left(1-\alpha \right)}{B(\alpha )}F_{1}\left(t,S\right)+\frac{\alpha }{B(\alpha )}\int_{0}^{t}F_{1}\left(\zeta ,S\right)d\zeta . \end{array}$$

For $t=t_{n+1}$, n=0,1,2,…, we obtain

(35) $$\begin{array}{ccc} S\left(t_{n+1}\right)-S_{0} & = & \frac{1-\alpha }{B(\alpha )}F_{1}\left(t_{n},S_{n}\right)+\frac{\alpha }{B(\alpha )}\int_{0}^{t_{n+1}}F_{1}\left(t,S\right)dt. \end{array}$$

The successive terms difference is given as follows:

(36) $$\begin{array}{ccc} S_{n+1}-S_{n} & = & \frac{1-\alpha }{B(\alpha )}\left\{F_{1}\left(t_{n},S_{n}\right)-F_{1}\left(t_{n-1},S_{n-1}\right)\right\}+\frac{\alpha }{B(\alpha )}\int_{t_{n}}^{t_{n+1}}F_{1}\left(t,S\right)dt. \end{array}$$

Over the close interval $\left[t_{k},t_{\left(k+1\right)}\right]$, the function $F_{1}\left(t,S\right)$ can be approximated by the interpolation polynomial

(37) $$\begin{array}{ccc} P_{k}\left(t\right) & ≅ & \frac{f(t_{k},y_{k})}{h}\left(t-t_{k-1}\right)-\frac{f(t_{k-1},y_{k-1}).}{h}\left(t-t_{k}\right), \end{array}$$

where $h=t_{n}-t_{n-1}.$ Calculating the integral in Equation (36) using above polynomial approximation we get

(38) $$\begin{array}{ccc} \int_{t_{n}}^{t_{n+1}}F_{1}\left(t,S\right)dt & = & \int_{t_{n}}^{t_{n+1}}\frac{F_{1}\left(t_{n},S_{n}\right)}{h}\left(t-t_{n-1}\right)-\frac{F_{1}\left(t_{n-1},S_{n-1}\right)}{h}\left(t-t_{n}\right)dt\\ & & =\frac{3h}{2}F_{1}\left(t_{n},S_{n}\right)-\frac{h}{2}F_{1}\left(t_{n-1},S_{n-1}\right). \end{array}$$

Substituting Equation (38) in (36) and after simplification we obtain

(39) $$\begin{array}{ccc} S_{n+1} & = & S_{n}+\left(\frac{1-\alpha }{B(\alpha )}+\frac{3h}{2B(\alpha )}\right)F_{1}\left(t_{n},S_{n}\right)-\left(\frac{1-\alpha }{B(\alpha )}+\frac{\alpha h}{2B(\alpha )}\right)F_{1}\left(t_{n-1},S_{n-1}\right). \end{array}$$

In a similar way, for the rest of equations of system Equation (33) we obtain the recursive formula as below

(40) $$\begin{array}{ccc} I_{n+1} & = & I_{0}+\left(\frac{1-\alpha }{B(\alpha )}+\frac{3h}{2B(\alpha )}\right)F_{2}\left(t_{n},I_{n}\right)-\left(\frac{1-\alpha }{B(\alpha )}+\frac{\alpha h}{2B(\alpha )}\right)F_{2}\left(t_{n-1},I_{n-1}\right),\\ R_{n+1} & = & R_{0}+\left(\frac{1-\alpha }{B(\alpha )}+\frac{3h}{2B(\alpha )}\right)F_{3}\left(t_{n},R_{n}\right)-\left(\frac{1-\alpha }{B(\alpha )}+\frac{\alpha h}{2B(\alpha )}\right)F_{3}\left(t_{n-1},R_{n-1}\right),\\ D_{n+1} & = & D_{0}+\left(\frac{1-\alpha }{B(\alpha )}+\frac{3h}{2B(\alpha )}\right)F_{4}\left(t_{n},D_{n}\right)-\left(\frac{1-\alpha }{B(\alpha )}+\frac{\alpha h}{2B(\alpha )}\right)F_{4}\left(t_{n-1},D_{n-1}\right),\\ P_{n+1} & = & P_{0}+\left(\frac{1-\alpha }{B(\alpha )}+\frac{3h}{2B(\alpha )}\right)F_{5}\left(t_{n},P_{n}\right)-\left(\frac{1-\alpha }{B(\alpha )}+\frac{\alpha h}{2B(\alpha )}\right)F_{5}\left(t_{n-1},P_{n-1}\right). \end{array}$$

**Figure 8:**
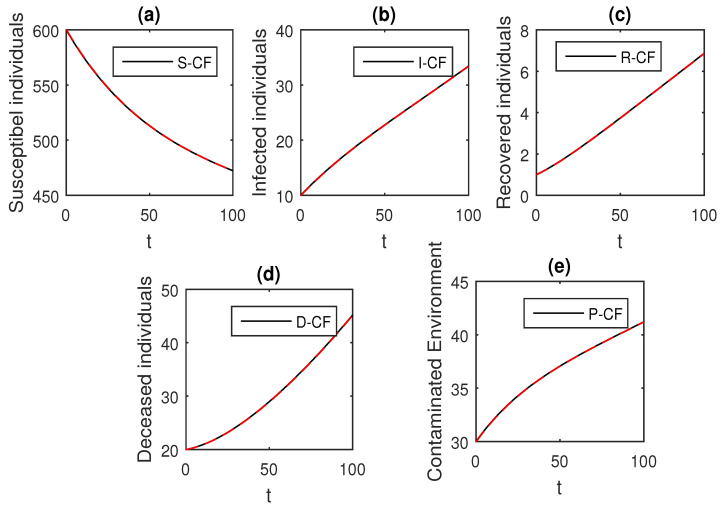
The graphical results show the dynamics of the Caputo–Fabrizio model (33), when α=1, where (**a**) Susceptible individuals, (**b**) Infected individuals, (**c**) recovered individuals, (**d**) deceased individuals, (**e**) Environment pathogens.

**Figure 9:**
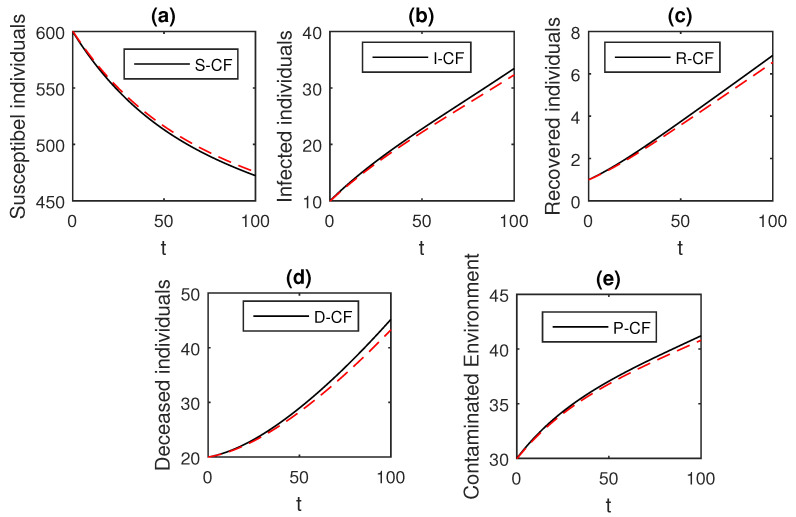
The graphical results show the dynamics of the Caputo–Fabrizio model (33), when α=0.95, where (**a**) Susceptible individuals, (**b**) Infected individuals, (**c**) recovered individuals, (**d**) deceased individuals, (**e**) Environment pathogens.

**Figure 10:**
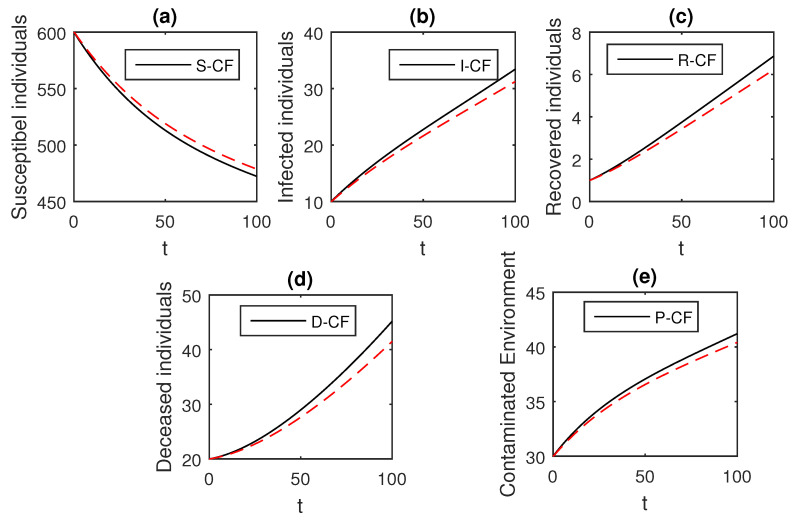
The graphical results show the dynamics of the Caputo–Fabrizio model (33), when α=0.9, where (**a**) Susceptible individuals, (**b**) Infected individuals, (**c**) recovered individuals, (**d**) deceased individuals, (**e**) Environment pathogens.

**Figure 11:**
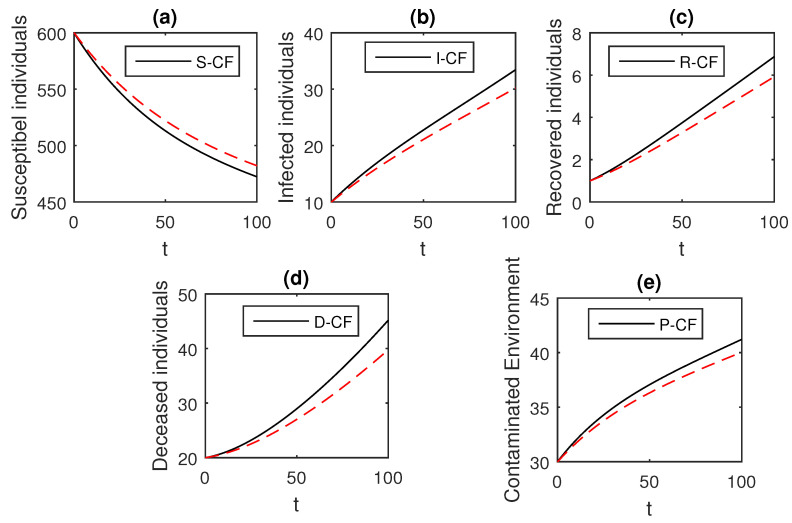
The graphical results show the dynamics of the Caputo–Fabrizio model (33), when α=0.85, where (**a**) Susceptible individuals, (**b**) Infected individuals, (**c**) recovered individuals, (**d**) deceased individuals, (**e**) Environment pathogens.

**Figure 12:**
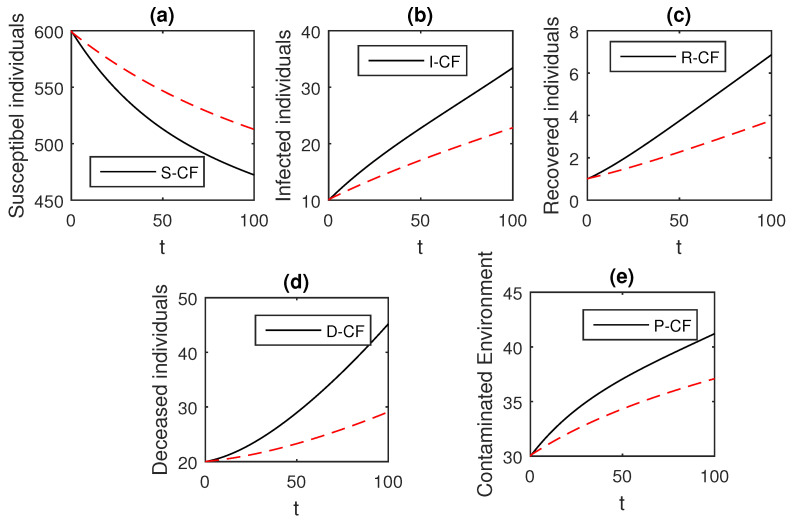
The graphical results show the dynamics of the Caputo–Fabrizio model (33), when α=0.5, where (**a**) Susceptible individuals, (**b**) Infected individuals, (**c**) recovered individuals, (**d**) deceased individuals, (**e**) Environment pathogens.

**Figure 13:**
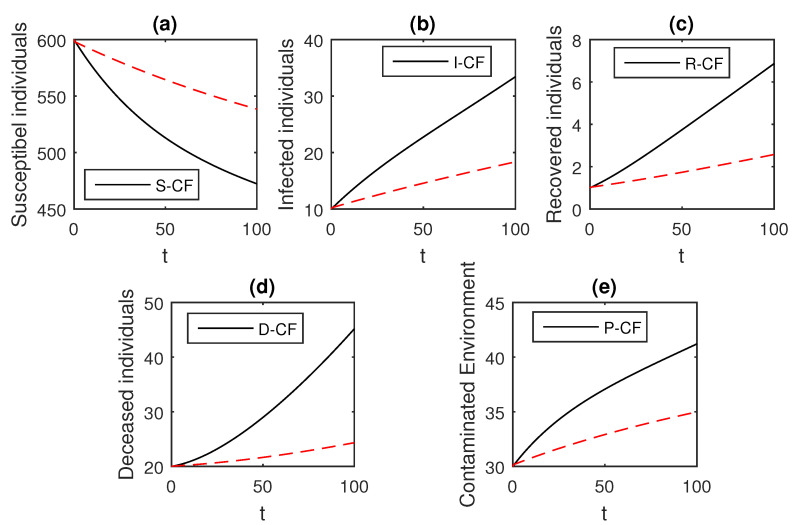
The graphical results show the dynamics of the Caputo–Fabrizio model (33), when α=0.3, where (**a**) Susceptible individuals, (**b**) Infected individuals, (**c**) recovered individuals, (**d**) deceased individuals, (**e**) Environment pathogens.

**Figure 14:**
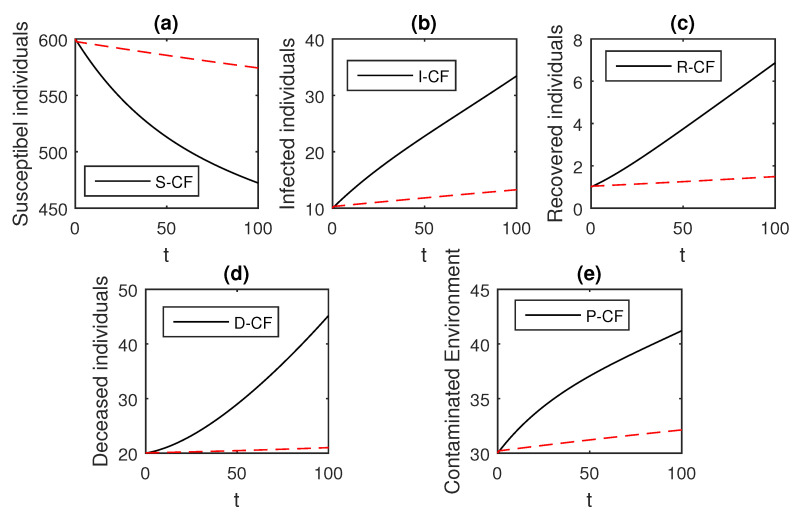
The graphical results show the dynamics of the Caputo–Fabrizio model (33), when α=0.1, where (**a**) Susceptible individuals, (**b**) Infected individuals, (**c**) recovered individuals, (**d**) deceased individuals, (**e**) Environment pathogens.

The numerical scheme presented above by considering the parameter values, d=0.05, δ=0.05, ϕ=0.06, Λ=10, ϵ=0.008, ξ=0.004, κ=0.03, ψ=0.01, $\beta_{1}=0.006$, $\beta_{2}=0.012$, ϖ=1, and θ=0.004, we have the graphical results for the Ebola disease model in Caputo–Fabrizio model Equation (33). Various graphical results considering the fractional order parameter α are presented, see Figure 8, Figure 9, Figure 10, Figure 11, Figure 12, Figure 13 and Figure 14. In these figures, we obtain various graphical results for α, and we observe that by decreasing the value of α the infected compartments are decreasing well. Especially, when choosing α=0.3,0.1, we can see that the number of infected individuals decreases rapidly.

### 4.6. Ebola Model in the Atangana–Baleanu Sense

We can express the model given by Equation (14) in Atangana–Baleanu derivative as follows:

(41) $$\left\{\begin{array}{ccc} {}_{0}^{ABC}D_{t}^{\alpha }S & = & \Lambda -\lambda S-dS,\\ {}_{0}^{ABC}D_{t}^{\alpha }I & = & \lambda S-(d+\delta +\phi_{1})I,\\ {}_{0}^{ABC}D_{t}^{\alpha }R & = & \phi_{1}I-dR,\\ {}_{0}^{ABC}D_{t}^{\alpha }D & = & (d+\delta )I-\varepsilon D,\\ {}_{0}^{ABC}D_{t}^{\alpha }P & = & \varpi +\xi I+\theta D-\kappa P, \end{array}\right.$$

where $\lambda =\beta_{1}I+\beta_{2}D+\psi P$.

### 4.7. Existence of Solutions for the Atangana–Baleanu Model

It is obvious that the given model Equation (14) shows the dynamics of Ebola disease, which is described by a nonlinear system of differential equations, so it is not possible to obtain their exact solution but the existence of an approximate solution can be very effective if we show that the solution for the Ebola disease model Equation (41) under some conditions exists. To do this, we follow the results of fixed point theory for the given Ebola disease model Equation (41). We write the Ebola disease model given by Equation (41) for simplification purposes as follows:

(42) $$\begin{array}{c} \left\{\begin{array}{c} {}_{0}^{ABC}D_{t}^{\alpha }x\left(t\right)=F\left(t,x\left(t\right)\right),\\ x\left(0\right)=x_{0},\;\;0<t<T<\infty , \end{array}\right. \end{array}$$

where x(t)=(S,I,R,D,P) represent the vector with state variables S,I,R,D,P and is a continuous vector function and can be defined as follows:

(43) $$\begin{array}{c} F=\left(\begin{array}{c} F_{1}\\ F_{2}\\ F_{3}\\ F_{4}\\ F_{5} \end{array}\right)=\left(\begin{array}{c} \Lambda -\lambda S-dS\\ \lambda S-(d+\delta +\phi_{1})I\\ \phi_{1}I-dR\\ (d+\delta )I-\varepsilon D\\ \varpi +\xi I+\theta D-\kappa P \end{array}\right). \end{array}$$

The function F can be shown easily to satisfy the Lipschitz condition and can be represented as:

(44) $$\begin{array}{ccc} ∥F\left(t,x_{1}\left(t\right)\right)-F\left(t,x_{2}\left(t\right)\right)∥ & \leq & K∥x_{1}\left(t\right)-x_{2}\left(t\right)∥. \end{array}$$

Now we have the results for the existence and uniqueness for the Ebola disease model in the Atangana–Baleanu derivative sense. We state and prove the following theorem:

**Theorem** **3.** *The Ebola disease model in the Atangana–Baleanu form Equation (41) can have a unique solution under some conditions if the following holds*

(45) $$\frac{\left(1-\alpha \right)}{ABC(\alpha )}K+\alpha \frac{T_{max}^{\alpha }}{ABC(\alpha )\Gamma (\alpha )}K<1.$$

**Proof.**  The use of the Atangana–Baleanu fractional integration on model Equation (42) both sides, the following is obtained,

(46) $$\begin{array}{c} x\left(t\right)=x_{0}+\frac{1-\alpha }{ABC(\alpha )}F\left(t,x\left(t\right)\right)+\frac{\alpha }{ABC(\alpha )\Gamma (\alpha )}\int_{0}^{t}{\left(t-\eta \right)}^{\alpha -1}F\left(\eta ,x\left(\eta \right)\right)d\eta . \end{array}$$

Suppose J=(0,T) and the operator $Y:C\left(J,R^{5}\right)\to C\left(J,R^{5}\right)$ defined by

(47) $$\begin{array}{c} Y\left[x\left(t\right)\right]=x_{0}+\frac{1-\alpha }{ABC(\alpha )}F\left(t,x\left(t\right)\right)+\frac{\alpha }{ABC(\alpha )\Gamma (\alpha )}\int_{0}^{t}{\left(t-\eta \right)}^{\alpha -1}F\left(\eta ,x\left(\eta \right)\right)d\eta . \end{array}$$

Then we can write Equation (46) as follows:

(48) $$\begin{array}{c} x(t)=Y[x(t)]. \end{array}$$

We have, after applying the supremum norm on *J*,

(49) $$\begin{array}{c} {∥x\left(t\right)∥}_{J}={sup}_{t\in J}∥x\left(t\right)∥,\;\;x\left(t\right)\in C. \end{array}$$

Obviously, $C(J,R^{5})$ and the norm ${∥.∥}_{J}$ is a Banach space. Using the operator Equation (48), the following is presented

(50) $$\begin{array}{ccc} ∥Y\left[x_{1}\left(t\right)\right]-Y\left[x_{2}\left(t\right)\right]{∥}_{J} & \leq & ∥\frac{\left(1-\alpha \right)}{ABC(\alpha )}(F\left(t,x_{1}\left(t\right)\right)-F\left(t,x_{2}\left(t\right)\right)+\frac{\alpha }{ABC(\alpha )\Gamma (\alpha )}\times \\ & & \int_{0}^{t}{\left(t-\eta \right)}^{\alpha -1}\left(F\left(\eta ,x_{1}\left(\eta \right)\right)-F\left(\eta ,x_{2}\left(\eta \right)\right)\right)d\eta {∥}_{J}. \end{array}$$

Using the triangular inequality and Lipschitz condition presented in Equation (44) with some simplifications, we have

(51) $$\begin{array}{ccc} ∥Y\left[x_{1}\left(t\right)\right]-Y\left[x_{1}\left(t\right)\right]{∥}_{J} & \leq & \left(\frac{(1-\alpha )K}{ABC(\alpha )}+\frac{\alpha }{ABC(\alpha )\Gamma (\alpha )}KT^{\alpha }\right){∥x_{1}\left(t\right)-x_{2}\left(t\right)∥}_{J}. \end{array}$$

Finally, we have

(52) $$\begin{array}{ccc} ∥Y\left[x_{1}\left(t\right)\right]-Y\left[x_{1}\left(t\right)\right]{∥}_{J} & \leq & L∥x_{1}\left(t\right)-x_{2}{\left(t\right)∥}_{J}, \end{array}$$

where

$$L=\frac{(1-\alpha )M}{ABC(\alpha )}+\frac{\alpha }{ABC(\alpha )\Gamma (\alpha )}MT^{\alpha }.$$

If the condition given by Equation (45) holds then the operator Y will be a contraction. Thus, the Banach fixed point theorem ensures that a unique solution for the Ebola disease model in the Atangana–Baleanu form Equation (41) exists, Equation (42). □

### 4.8. Numerical Results for the Atangana–Baleanu Model and Simulation Results

In the present subsection we aim to obtain the numerical results for the Ebola disease model in the Atangana–Baleanu form given by Equation (41). First, we provide a numerical scheme in details and then show the graphical results for various values of the fractional order parameter α. The scheme given in [28] will be used to obtain the approximate solution of the Ebola disease model in the Atangana–Baleanu form Equation (41).

We write the model Equation (42) after using the fundamental theorem of fractional calculus:

(53) $$\begin{array}{ccc} w(t)-w(0) & = & \frac{\left(1-\alpha \right)}{ABC(\alpha )}F\left(t,w\left(t\right)\right)+\frac{\alpha }{ABC(\alpha )\times \Gamma (\alpha )}\int_{0}^{t}F\left(\phi ,x\left(\phi \right)\right){\left(t-\phi \right)}^{\alpha -1}d\phi . \end{array}$$

At $t=t_{n+1}$, n=0,1,2,…, we have

(54) $$\begin{array}{ccc} w\left(t_{n+1}\right)-w\left(0\right) & = & \frac{1-\alpha }{ABC(\alpha )}F\left(t_{n},w\left(t_{n}\right)\right)+\\ & & \frac{\alpha }{ABC(\alpha )\times \Gamma (\alpha )}\int_{0}^{t_{n+1}}F\left(\phi ,w\left(\phi \right)\right){\left(t_{n+1}-\phi \right)}^{\alpha -1}d\phi ,\\ & = & \frac{1-\alpha }{ABC(\alpha )}F\left(t_{n},w\left(t_{n}\right)\right)+\\ & & \frac{\alpha }{ABC(\alpha )\times \Gamma (\alpha )}\sum_{j=0}^{n}\int_{t_{j}}^{t_{j+1}}F\left(\phi ,w\left(\phi \right)\right){\left(t_{n+1}-\phi \right)}^{\alpha -1}d\phi . \end{array}$$

The function F(ϕ,w(ϕ)) can be approximated over $\left[t_{j},t_{j+1}\right]$, using the interpolation polynomial

(55) $$\begin{array}{ccc} F(\phi ,w(\phi )) & ≅ & \frac{F(t_{j},w\left(t_{j}\right))}{h}\left(t-t_{j-1}\right)-\frac{F(t_{j-1},w\left(t_{j-1}\right))}{h}\left(t-t_{j}\right). \end{array}$$

Substituting in Equation (54) we get

(56) $$\begin{array}{ccc} w(t_{n+1}) & = & w\left(0\right)+\frac{1-\alpha }{ABC(\alpha )}F\left(t_{n},w\left(t_{n}\right)\right)+\\ & & \frac{\alpha }{ABC(\alpha )\times \Gamma (\alpha )}\sum_{j=0}^{n}(\frac{F(t_{j},w\left(t_{j}\right))}{h}\int_{t_{j}}^{t_{j+1}}\left(t-t_{j-1}\right){\left(t_{n+1}-t\right)}^{\alpha -1}dt\\ & & -\frac{F(t_{j-1},w\left(t_{j-1}\right))}{h}\int_{t_{j}}^{t_{j+1}}\left(t-t_{j}\right){\left(t_{n+1}-t\right)}^{\alpha -1}dt). \end{array}$$

After some calculation, we obtain the following:

(57) $$\begin{array}{ccc} w(t_{n+1}) & = & w\left(t_{0}\right)+\frac{1-\alpha }{ABC(\alpha )}F\left(t_{n},w\left(t_{n}\right)\right)+\\ & & \frac{\alpha }{ABC(\alpha )}\sum_{j=0}^{n}\\ & & (\frac{h^{\alpha }F\left(t_{j},w\left(t_{j}\right)\right)}{\Gamma (\alpha +2)}\left({\left(n+1-j\right)}^{\alpha }\left(n-j+2+\alpha \right)-{\left(n-j\right)}^{\alpha }\left(n-j+2+2\alpha \right)\right)\\ & & -\frac{h^{\alpha }F\left(t_{j-1},w\left(t_{j-1}\right)\right)}{\Gamma (\alpha +2)}\left({\left(n+1-j\right)}^{\alpha +1}-{\left(n-j\right)}^{\alpha }\left(n-j+1+\alpha \right)\right)). \end{array}$$

For the Ebola disease model we have the following results:

$$\begin{array}{ccc} S(t_{n+1}) & = & S\left(t_{0}\right)+\frac{1-\alpha }{ABC(\alpha )}F_{1}\left(t_{n},w\left(t_{n}\right)\right)+\\ & & \frac{\alpha }{ABC(\alpha )}\sum_{j=0}^{n}\\ & & (\frac{h^{\alpha }F_{1}\left(t_{j},w\left(t_{j}\right)\right)}{\Gamma (\alpha +2)}\left({\left(n+1-j\right)}^{\alpha }\left(n-j+2+\alpha \right)-{\left(n-j\right)}^{\alpha }\left(n-j+2+2\alpha \right)\right)\\ & & -\frac{h^{\alpha }F_{1}\left(t_{j-1},w\left(t_{j-1}\right)\right)}{\Gamma (\alpha +2)}\left({\left(n+1-j\right)}^{\alpha +1}-{\left(n-j\right)}^{\alpha }\left(n-j+1+\alpha \right)\right)), \end{array}$$

$$\begin{array}{ccc} I(t_{n+1}) & = & I\left(t_{0}\right)+\frac{1-\alpha }{ABC(\alpha )}F_{2}\left(t_{n},w\left(t_{n}\right)\right)+\\ & & \frac{\alpha }{ABC(\alpha )}\sum_{j=0}^{n}\\ & & (\frac{h^{\alpha }F_{2}\left(t_{j},w\left(t_{j}\right)\right)}{\Gamma (\alpha +2)}\left({\left(n+1-j\right)}^{\alpha }\left(n-j+2+\alpha \right)-{\left(n-j\right)}^{\alpha }\left(n-j+2+2\alpha \right)\right)\\ & & -\frac{h^{\alpha }F_{2}\left(t_{j-1},w\left(t_{j-1}\right)\right)}{\Gamma (\alpha +2)}\left({\left(n+1-j\right)}^{\alpha +1}-{\left(n-j\right)}^{\alpha }\left(n-j+1+\alpha \right)\right)), \end{array}$$

$$\begin{array}{ccc} R(t_{n+1}) & = & R\left(t_{0}\right)+\frac{1-\alpha }{ABC(\alpha )}F_{3}\left(t_{n},w\left(t_{n}\right)\right)+\\ & & \frac{\alpha }{ABC(\alpha )}\sum_{j=0}^{n}\\ & & (\frac{h^{\alpha }F_{3}\left(t_{j},w\left(t_{j}\right)\right)}{\Gamma (\alpha +2)}\left({\left(n+1-j\right)}^{\alpha }\left(n-j+2+\alpha \right)-{\left(n-j\right)}^{\alpha }\left(n-j+2+2\alpha \right)\right)\\ & & -\frac{h^{\alpha }F_{3}\left(t_{j-1},w\left(t_{j-1}\right)\right)}{\Gamma (\alpha +2)}\left({\left(n+1-j\right)}^{\alpha +1}-{\left(n-j\right)}^{\alpha }\left(n-j+1+\alpha \right)\right)), \end{array}$$

$$\begin{array}{ccc} D(t_{n+1}) & = & D\left(t_{0}\right)+\frac{1-\alpha }{ABC(\alpha )}F_{4}\left(t_{n},w\left(t_{n}\right)\right)+\\ & & \frac{\alpha }{ABC(\alpha )}\sum_{j=0}^{n}\\ & & (\frac{h^{\alpha }F_{4}\left(t_{j},w\left(t_{j}\right)\right)}{\Gamma (\alpha +2)}\left({\left(n+1-j\right)}^{\alpha }\left(n-j+2+\alpha \right)-{\left(n-j\right)}^{\alpha }\left(n-j+2+2\alpha \right)\right)\\ & & -\frac{h^{\alpha }F_{4}\left(t_{j-1},w\left(t_{j-1}\right)\right)}{\Gamma (\alpha +2)}\left({\left(n+1-j\right)}^{\alpha +1}-{\left(n-j\right)}^{\alpha }\left(n-j+1+\alpha \right)\right)), \end{array}$$

(58) $$\begin{array}{ccc} P(t_{n+1}) & = & P\left(t_{0}\right)+\frac{1-\alpha }{ABC(\alpha )}F_{5}\left(t_{n},w\left(t_{n}\right)\right)+\\ & & \frac{\alpha }{ABC(\alpha )}\sum_{j=0}^{n}\\ & & (\frac{h^{\alpha }F_{5}\left(t_{j},w\left(t_{j}\right)\right)}{\Gamma (\alpha +2)}\left({\left(n+1-j\right)}^{\alpha }\left(n-j+2+\alpha \right)-{\left(n-j\right)}^{\alpha }\left(n-j+2+2\alpha \right)\right)\\ & & -\frac{h^{\alpha }F_{5}\left(t_{j-1},w\left(t_{j-1}\right)\right)}{\Gamma (\alpha +2)}\left({\left(n+1-j\right)}^{\alpha +1}-{\left(n-j\right)}^{\alpha }\left(n-j+1+\alpha \right)\right)). \end{array}$$

**Figure 15:**
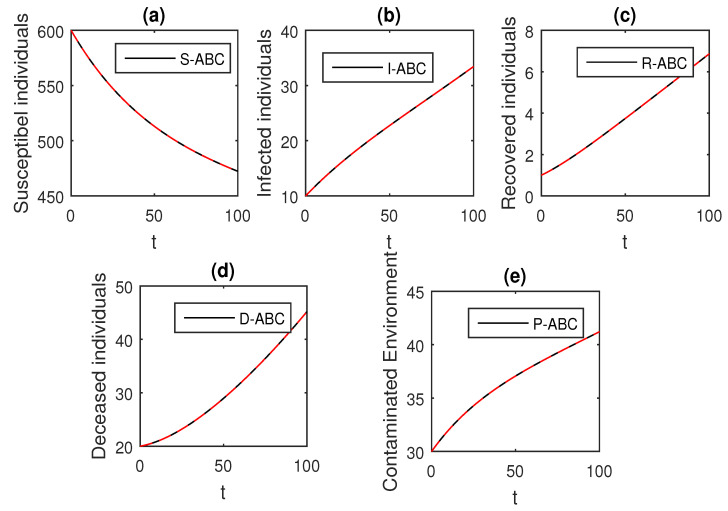
The graphical results show the dynamics of the Atangana–Baleanu model (41), when α=1, where (**a**) Susceptible individuals, (**b**) Infected individuals, (**c**) recovered individuals, (**d**) deceased individuals, (**e**) Environment pathogens.

**Figure 16:**
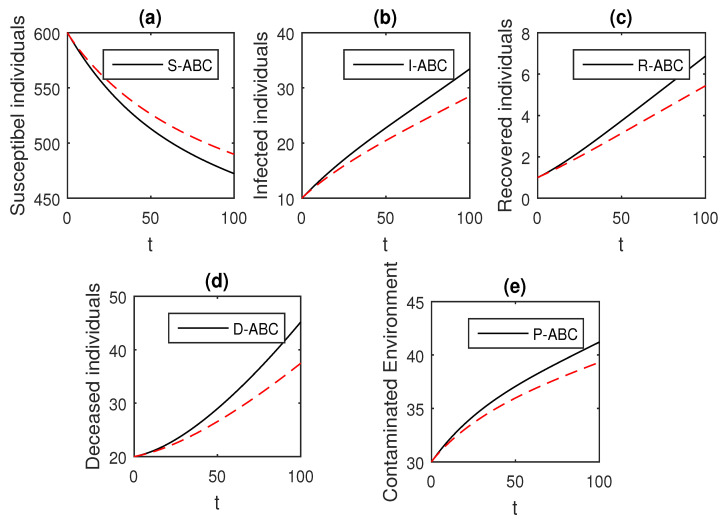
The graphical results show the dynamics of the Atangana–Baleanu model (41), when α=0.95, where (**a**) Susceptible individuals, (**b**) Infected individuals, (**c**) recovered individuals, (**d**) deceased individuals, (**e**) Environment pathogens.

**Figure 17:**
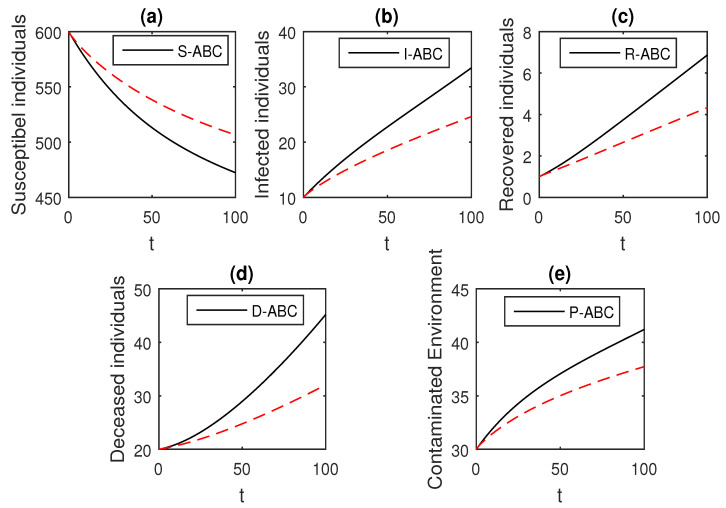
The graphical results show the dynamics of the Atangana–Baleanu model (41), when α=0.9, where (**a**) Susceptible individuals, (**b**) Infected individuals, (**c**) recovered individuals, (**d**) deceased individuals, (**e**) Environment pathogens.

**Figure 18:**
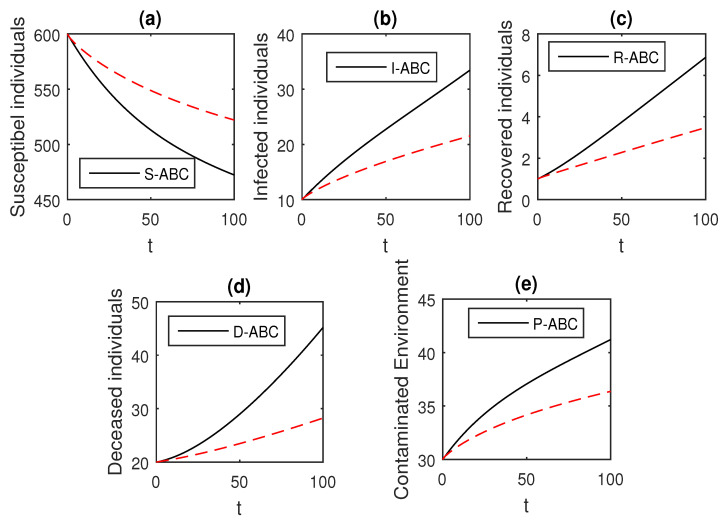
The graphical results show the dynamics of the Atangana–Baleanu model (41), when α=0.85, where (**a**) Susceptible individuals, (**b**) Infected individuals, (**c**) recovered individuals, (**d**) deceased individuals, (**e**) Environment pathogens.

**Figure 19:**
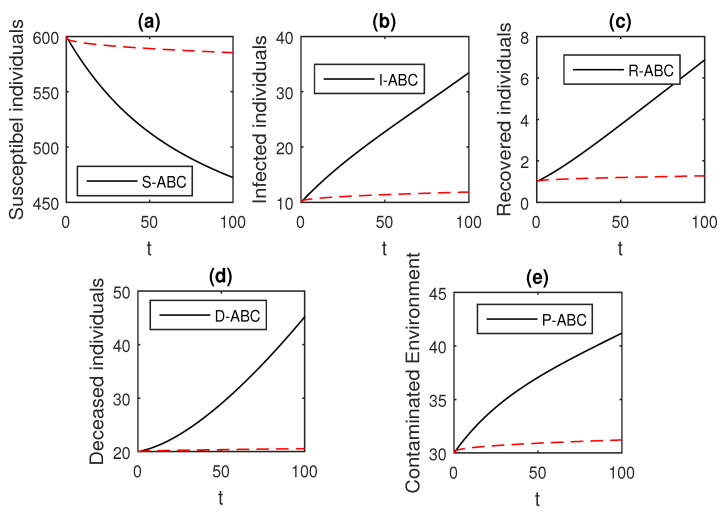
The graphical results show the dynamics of the Atangana–Baleanu model (41), when α=0.5, where (**a**) Susceptible individuals, (**b**) Infected individuals, (**c**) recovered individuals, (**d**) deceased individuals, (**e**) Environment pathogens.

**Figure 20:**
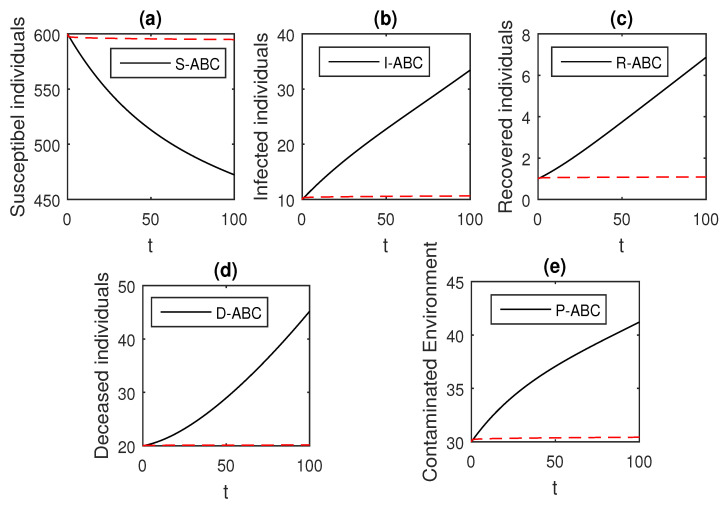
The graphical results show the dynamics of the Atangana–Baleanu model (41), when α=0.3, where (**a**) Susceptible individuals, (**b**) Infected individuals, (**c**) recovered individuals, (**d**) deceased individuals, (**e**) Environment pathogens.

**Figure 21:**
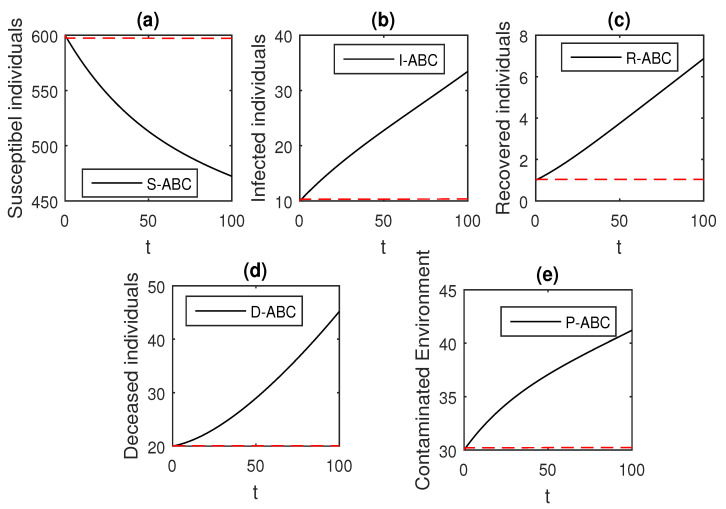
The graphical results show the dynamics of the Atangana–Baleanu model (41), when α=0.1, where (**a**) Susceptible individuals, (**b**) Infected individuals, (**c**) recovered individuals, (**d**) deceased individuals, (**e**) Environment pathogens.

We using the above scheme for the numerical solution of the Ebola disease model Equation (41) and obtain the graphical results shown in Figure 15, Figure 16, Figure 17, Figure 18, Figure 19, Figure 20 and Figure 21 by considering different values of the fractional order parameter α. In these Figure 15, Figure 16, Figure 17, Figure 18, Figure 19, Figure 20 and Figure 21, by decreasing the values of the fractional order parameter α, the population of infected compartments decreases more efficiently for the cases of α=0.3,0.1. One can see that the numerical results in the form of graphs obtained through the Atangana–Baleanu operator in comparison to the Caputo and Caputo–Fabrizio operator decrease the infection faster. So, from the above graphical results, it is suggested that the Atangana–Baleanu operator is more useful for infection elimination by decreasing the value of α.

### 4.9. Graphical Comparison of the Operators

**Figure 22:**
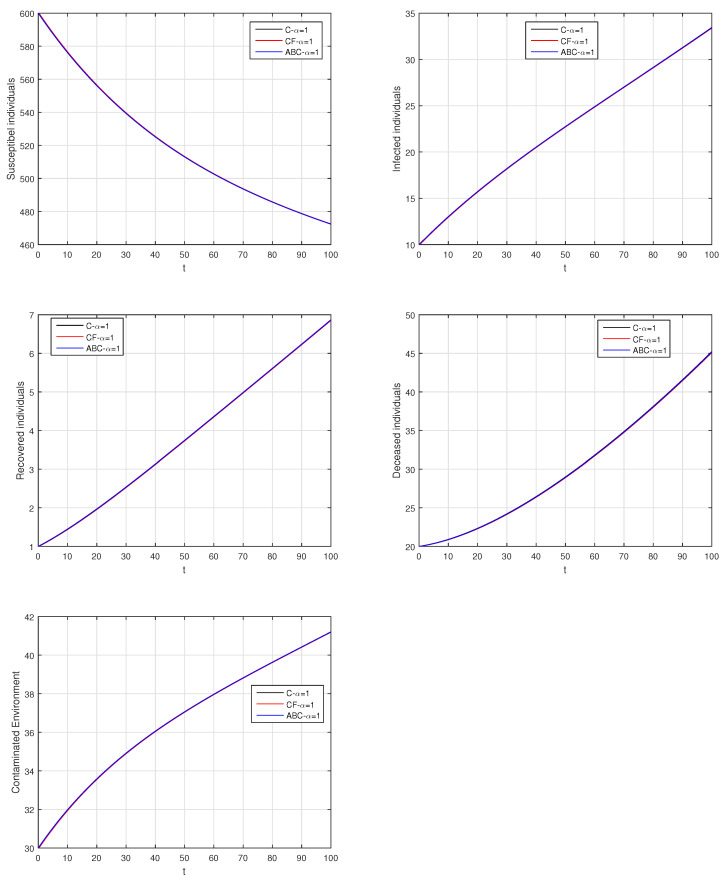
Comparison graphs for the Caputo, Caputo–Fabrizio, and the Atangana–Baleanu derivatives when α=1, where (**a**) Susceptible individuals, (**b**) Infected individuals, (**c**) recovered individuals, (**d**) deceased individuals, (**e**) Environment pathogens.

**Figure 23:**
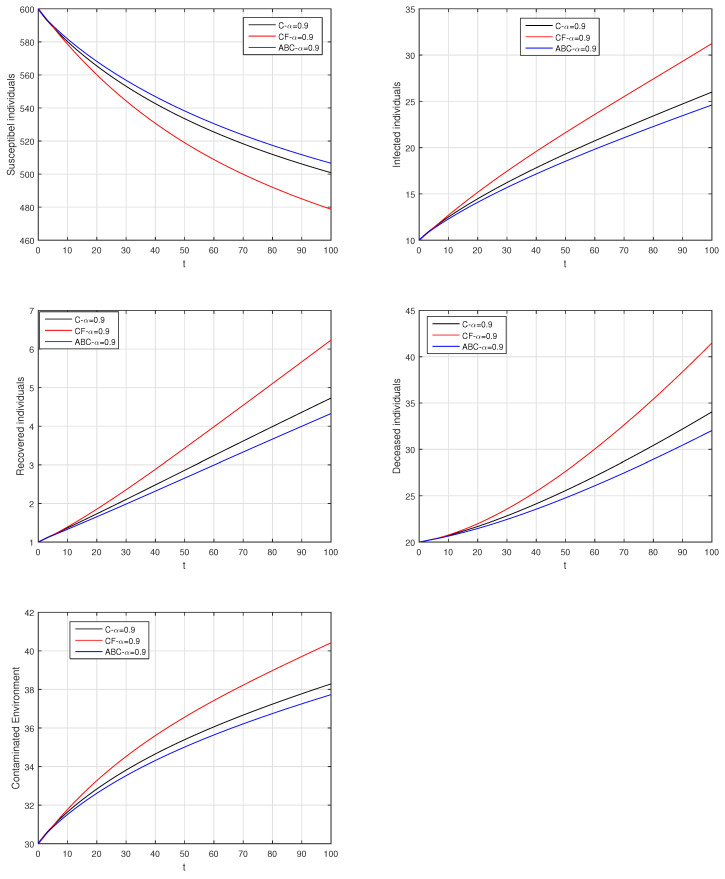
Comparison graphs for the Caputo, Caputo–Fabrizio, and the Atangana–Baleanu derivatives when α=0.9, where (**a**) Susceptible individuals, (**b**) Infected individuals, (**c**) recovered individuals, (**d**) deceased individuals, (**e**) Environment pathogens.

**Figure 24:**
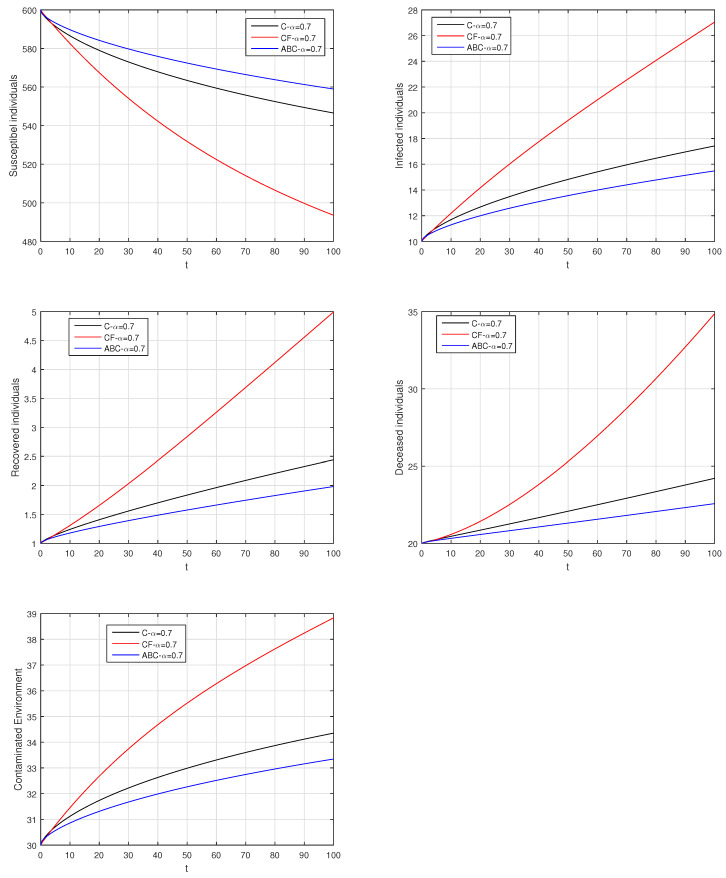
Comparison graphs for the Caputo, Caputo–Fabrizio, and the Atangana–Baleanu derivatives when α=0.7, where (**a**) Susceptible individuals, (**b**) Infected individuals, (**c**) recovered individuals, (**d**) deceased individuals, (e) Environment pathogens.

**Figure 25:**
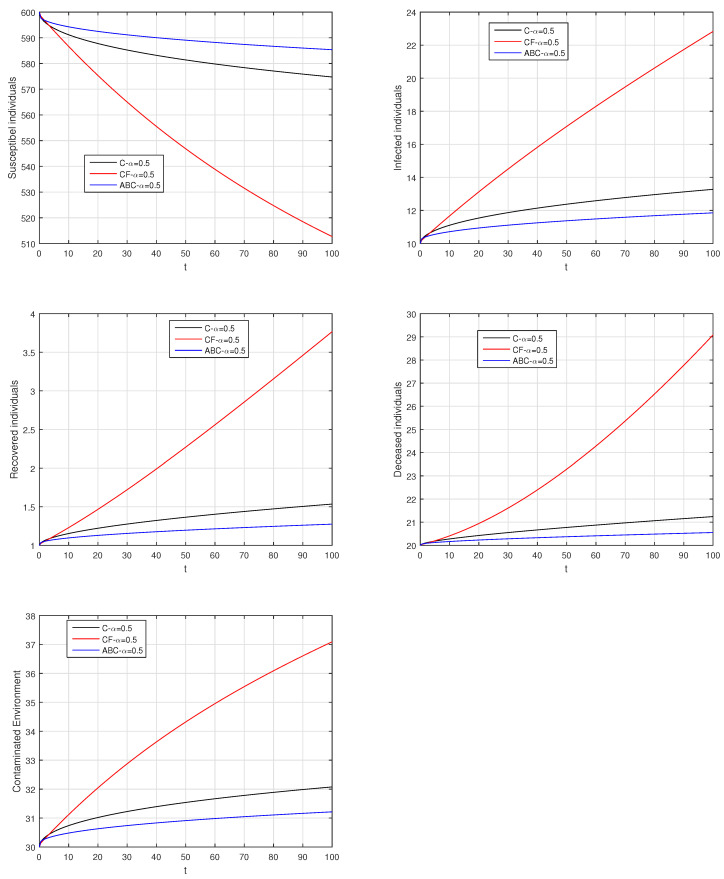
Comparison graphs for the Caputo, Caputo–Fabrizio, and the Atangana–Baleanu derivatives when α=0.5, where (**a**) Susceptible individuals, (**b**) Infected individuals, (**c**) recovered individuals, (**d**) deceased individuals, (**e**) Environment pathogens.

**Figure 26:**
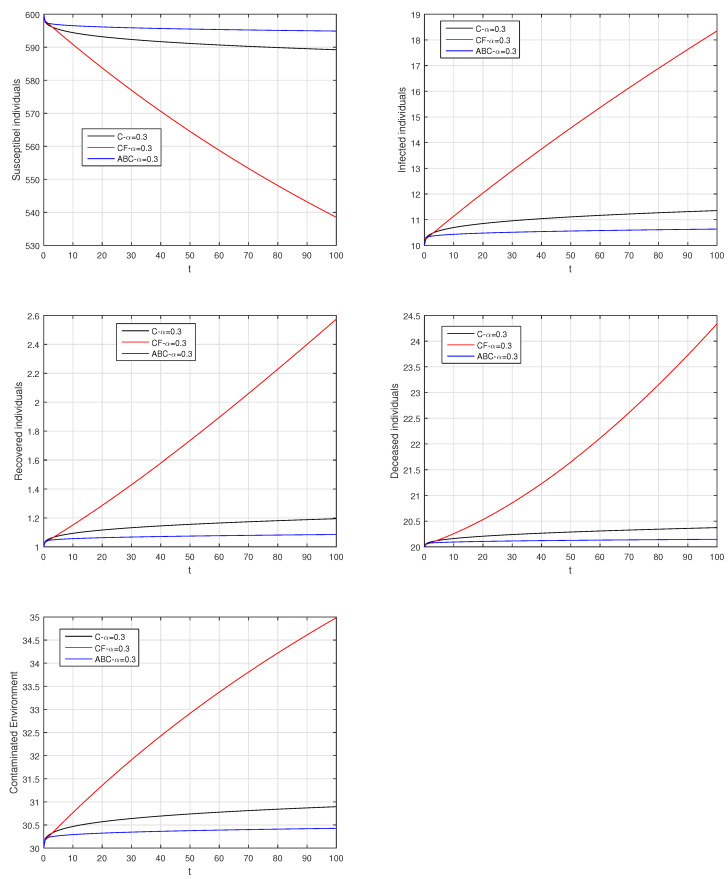
Comparison graphs for the Caputo, Caputo–Fabrizio, and the Atangana–Baleanu derivatives when α=0.3, where (**a**) Susceptible individuals, (**b**) Infected individuals, (**c**) recovered individuals, (**d**) deceased individuals, (**e**) Environment pathogens.

**Figure 27:**
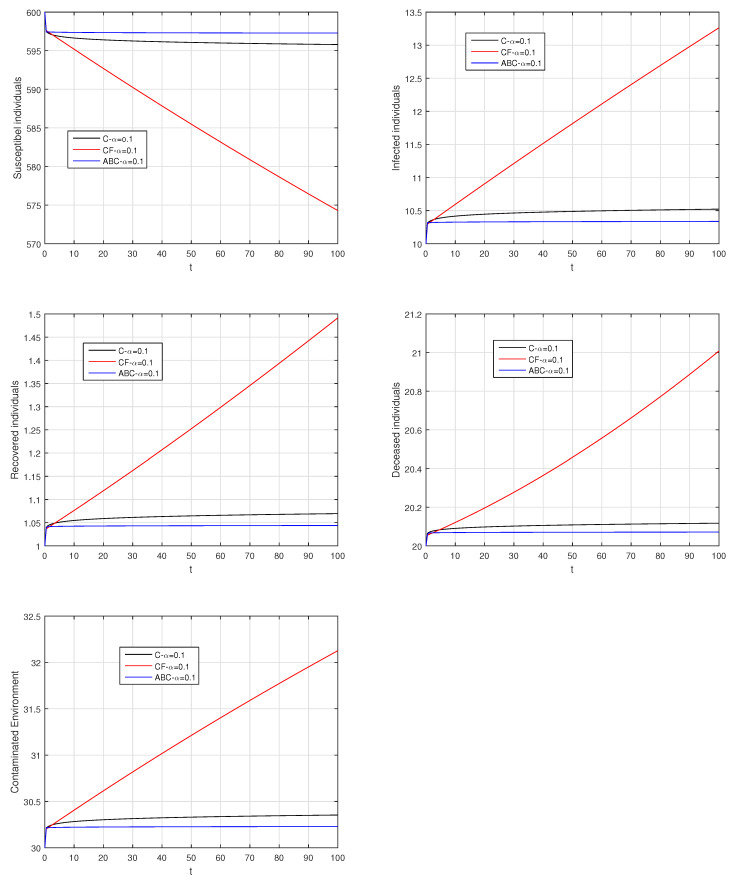
Comparison graphs for the Caputo, Caputo–Fabrizio, and the Atangana–Baleanu derivatives when α=0.1, where (**a**) Susceptible individuals, (**b**) Infected individuals, (**c**) recovered individuals, (**d**) deceased individuals, (**e**) Environment pathogens.

Here, we provide comparison plots for the Caputo, Caputo–Fabrizio, and the Atangana–Baleanu operators. We considered various values of the fractional order parameter α=1,0.9,0.7,0.5,0.3,0.1 and presented the graphical results for comparison (see Figure 22, Figure 23, Figure 24, Figure 25, Figure 26 and Figure 27). By decreasing the value of α it can be seen that the number of infected individuals decreases well, compared to the Caputo–Fabrizio and Caputo derivatives. Especially, for the cases when α=0.3,0.1, the Atangana–Baleanu derivative provides useful results for the infection elimination in comparison to the Caputo and the Caputo–Fabrizio operators.

## 5. Conclusions

We presented the dynamics of an Ebola disease model in the framework of fractional calculus. We applied three fractional operators, which are the Caputo, Caputo–Fabrizio, and the Atangana–Baleanu models. Initially, we proposed an epidemic model available literature for Ebola disease and then applied the proposed operators. The Ebola disease model with the Caputo derivative is presented and an effective numerical scheme for the numerical solution was provided. We used many values for the fractional order parameters and obtained the graphical results. The same model is used further and applied to the Caputo–Fabrizio derivative and we then presented a numerical solution for their solution. The solution was obtained and presented in graphical shape with the use of various fractional order parameter values. Then the newly established derivative known as the Atangana–Baleanu derivative was successfully applied to the Ebola disease model. The Ebola disease model in the Atangana–Baleanu sense is used and the uniqueness and existence were presented. Then, we presented a numerical scheme for the solution and presented various graphical results for α. Comparisons of the proposed three operators for various values of the fractional order parameter α=1,0.9,0.7,0.5,0.3,0.1 are presented and discussed. The comparison results show that the Atangana–Baleanu derivative is more helpful for disease elimination by decreasing the value of α, since the population of infected individuals decreased well. The use of three different fractional operators on the Ebola disease model suggests that the fractional order parameter greatly affects disease elimination for the non-integer case when decreasing α. Therefore, we suggest that the application of the various fractional derivatives on the present disease model shows the greater effectiveness of the arbitrary order derivative than that of the integer order model for the case of fractional order parameters.
